# A model for anterior vitrectomy in real patients: Simulation for practical training

**DOI:** 10.1038/s41433-024-02925-5

**Published:** 2024-01-18

**Authors:** Craig Wilde, Mary Awad, Alexander Foss, Georgios D. Panos, Harminder Dua

**Affiliations:** Department of Ophthalmology & Division of Ophthalmology and Vision Sciences, B Floor, EENT Centre, Queen’s Medical Centre, School of Medicine, University of Nottingham, Nottingham, UK

**Keywords:** Lens diseases, Surgery

Cataract surgery is the most common surgical procedure worldwide [1]. It is well established that posterior capsule rupture (PCR) is a complication that increases the risk of poor visual outcomes [2–4]. Anterior vitrectomy (AV) should be an essential skill with high priority within the training scheme, ensuring adequate exposure and documented competence. To achieve the certificate of completion of training (CCT) an arbitrary benchmark of 350 cataract surgeries is required in the UK. No such minimum number for AV is stipulated in the curriculum. We have demonstrated that cataract surgery training can be delivered while maintaining very low complication rates [5]. Consequently, it is increasingly recognised that senior trainees have limited exposure to cataract surgery complications, with a recent survey reporting only 9.1% felt confident in managing a PCR [6]. Among trainees who had performed over 350 cataract operations, the mean number of AV performed was low (3.5, range 1–7), providing minimal exposure to this complication, presenting a challenge in how to deliver adequate training. Only a minority (18.5%) felt they could manage PCR with vitreous loss independently [6], raising concerns that trainees lack practical experience and some may be inadequately prepared to deal with this complication upon starting their career as a consultant.

To date, there have been two proposed solutions to this problem:

- Use of simulation, which is becoming increasingly sophisticated and realistic.
- Cessation of the surgical procedure and prompt onward referral to vitreoretinal services for ongoing management.

With shortfalls in training and a lack of adequate exposure, the idea of a surgeon who can tackle anything and manage complications alone may be coming to an end. Given the rarity of this problem in skilled hands, closure of the eye and prompt referral is a strategy.

**Fig. 1:**
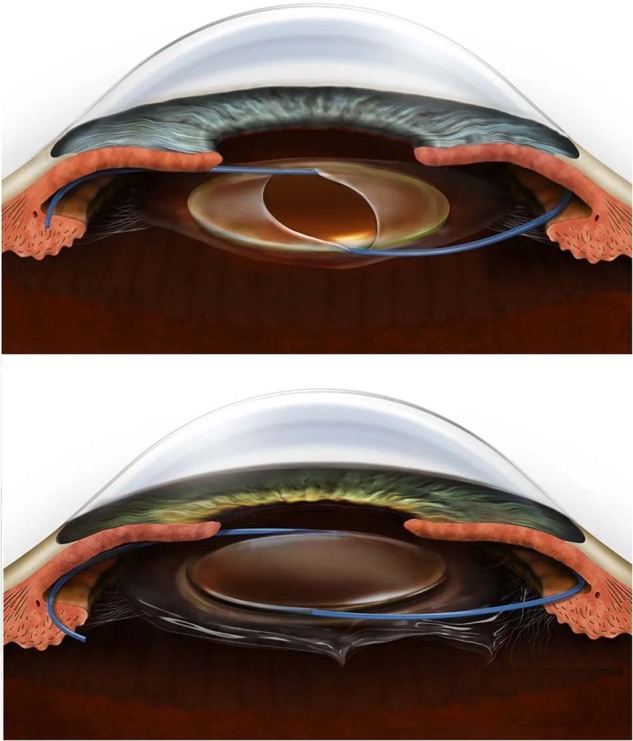
**Top:** IOL is positioned in the ciliary sulcus and the optic is captured posteriorly through the capsule opening**. Bottom:** IOL is positioned in the ciliary sulcus on top of a ruptured posterior capsule, the optic is NOT captured. (Figures are reproduced with permission from eyeillustrations.com).

We present a potential practical simulation in real patients that can be encouraged for all trainees, particularly those lacking confidence in performing AV or with inadequate hands-on exposure during training. The authors propose that during vitreoretinal (VR) surgery rotations trainees are encouraged to perform combined cataract surgery and removal of silicone oil using an anterior approach as described. Following the removal of the lens and cortex (using bimanual irrigation and aspiration) trainees should fill the bag with viscoelastic and perform a circular primary posterior capsulorhexis. There is inevitably residual retrolental vitreous hyaloid that is often intact and prevents adequate upward flow of silicone oil out of the eye when deploying the standard procedure of depressing the posterior lip of the cataract wound. Ordinarily, it can be punctured and disrupted with any instrument but its presence can be used as an opportunity for trainees to familiarise themselves with the process of AV. Residual hyaloid can be removed with a bimanual anterior vitrector to purposefully practice the important principles. It allows trainees to become familiar with instrument handling, appropriate settings and important principles, including disassociation of the infusion and cutter, turning the cutter on and off, directing the infusion into the chamber angle, using a low bottle height and high cut-rate and experiencing the effects of these actions. It allows trainees to familiarise themselves with bimanual AV, emphasising the importance of maintaining a closed anterior chamber and preservation of the anterior capsule. Once no residual hyaloid remains the trainee removes the silicone oil by increasing the infusion pressure and depressing the posterior lip of the cataract wound. Trainees then get the opportunity to practice the placement of a 3-piece sulcus intraocular lens implant with and without optic capture (Fig. 1). The instillation of miochol to constrict the pupil and the importance of checking for pupil peaking and wound integrity with a Weck-cell sponge can also be demonstrated and experienced.

Although the amount of vitreous is minimal and the model has limitations, it provides a safe in-house basic simulation using real instruments and lenses, allowing repeated practice as often as required in a controlled and stress-free environment. Trainees who have performed this simulation, with no previous practical AV experience have found it useful and we would like to share this model with the wider ophthalmic community as an option for addressing any shortfall in a trainee’s exposure to this important procedure. Considering that UK tertiary centres can realistically perform at least 2 to 3 silicone oil removals per month, the total number of procedures per semester (12–18) provides adequate exposure for trainees. However, the vitreoretinal rotation should be scheduled at a later stage of training (year 5 or above) to ensure the trainee is already a competent cataract surgeon and can fully benefit from the opportunity.

The authors recommend that the Royal College of Ophthalmologists in the UK and other bodies responsible for setting ophthalmology trainee curricula, introduce a formal strategy to improve AV exposure and training including the introduction of a minimum number as a requirement for this procedure and the need to be assessed to demonstrate competence. Practice by simulation such as with our model or others [7, 8] could form part of the curriculum. Strategies and guidance on how to best manage individuals, who reach the end of training with no personal experience in performing an AV, should be put in place.

After practicing with this model, during vitreoretinal rotations, senior trainees should be encouraged to perform (under supervision), complex cases with a high risk of vitreous loss, such as posterior polar and traumatic cataracts, those following accidental posterior capsular damage during intravitreal injections, and those with zonular weakness. The lack of AV training that currently prevails requires closure of the procedure and direct referral to a vitreoretinal surgeon. The delay so introduced, prolongs recovery time and adds to risks of post operative adverse events. The proposed model and modification to the training programme will help address the current lack of confidence and experience among trainees [6, 9], with better outcomes for patients.
